# Filling Roots with Gutta-Percha Dissolved in Chloroform

**Published:** 1893-04

**Authors:** R. I. Blakeman

**Affiliations:** New York


### FILLING ROOTS WITH GUTTA-PERCHA DISSOLVED IN CHLOROFORM.[1]

BY DR. R. I. BLAKEMAN, NEW YORK.

It has been suggested by a member of your Executive Committee that it might be of interest and possible assistance to some of the younger members of the profession to hear some testimony on the subject of filling roots with gutta-percha dissolved in chloroform. This solution, depending on the degree of fluidity, is very permeative. Perhaps some of you may have experienced getting it on your fingers; if so, you have doubtless noticed how it penetrates the fissures of the skin, and how difficult it is to remove at the time without the aid of a solvent. If an instrument be dipped into it, especially one that is a little rough, the gutta-percha adheres to it very closely, and remains so after the chloroform has evaporated. It sticks to the smooth surface of glass as well, and also to tooth-structure. And I might state here, though it does not directly bear on the subject, that it sometimes answers nicely to line gold with against which amalgam is to be placed, so that the gold may not be affected by the mercury. When the fluid is very thin, it seems as susceptible to capillary attraction as water. Therefore, as some of the canals we wish to fill are very fine, and we feel that they must be filled, as upon this largely depends the future welfare of the tooth, to fill them with this solution seems practical so far as the principles of physics are concerned. For the purpose of describing the process of manipulation, let us consider a molar, the roots of which must be filled from a posterior cavity difficult of access, a somewhat common occurrence. When the roots are dry and ready to fill, it is best to add some fresh chloroform to the solution kept on hand, so that the upper portion is quite thin, while the lower is left very thick. Then with a small broach, with a few fibres of cotton wrapped about the end, the solution can be carried to the canals, and, when the entrance to them is flooded over, it can be pumped in with a small bare broach.

After the canals are full of the thin solution, by dipping deeper into the supply the thicker gutta-percha is obtained, which can be pumped into the canals in like manner, the chloroform being worked out so that it can be evaporated with the chip-blower. If there should be a doubt as to the fluid having gone to the apex of any canal,

---

[1] Read before the New York Odontological Society, January 17, 1893.

it can be pushed farther by making a piston of warm gutta-percha. But great care should be taken in doing this, and the patient should be instructed to respond to the first sensation, for sufficient force may be brought to bear unconsciously to push the fluid through the foramen. When the canals are sufficiently large to permit of it, it is best to put in a gutta-percha point after they are full of the solution, but not so tight as to cause pressure at the end of the root. It might be well to emphasize this point, as any one not accustomed to filling roots in this way is very liable to force something through the apical foramen, which we all know the importance of avoiding in roots that have never had fistulous openings. My personal experience in following out this method is that I am more apt to do too much than not enough, and, after experiencing the results of going too far in some cases, find it easier to stop in time. It will be admitted, I think, that many valuable methods in practice are of additional value because they allow the operator, in case of any trouble, to easily undo his work, and it is on that basis that I claim this method of filling roots to be more advantageous than many others. I experienced the value of this method not very long ago in the following manner. A lower wisdom-tooth, buccal cavity, the root-canals of which were very fine, had been filled as above described. For reasons that have no bearing on the subject, it was decided that the filling should be removed from the roots. This was successfully accomplished by using mechanical means to remove all the gutta-percha accessible; then with a drop-syringe the pulp-chamber was filled with chloroform in order to dissolve the gutta-percha remaining in the roots. This was hastened by stirring it up with a broach. It was necessary to repeat this process only two or three times. I have brought with me for exhibition a bicuspid root which was filled in the mouth, and, owing to its being split, it gives us a chance to see how successful the operation was. The gold wire, twisted about the neck, was put there at the time of filling, for the purpose of holding the two pieces together, as the root was already split at that time.

